# OUTPUT: Choreographed and Reconfigured Human and Industrial Robot Bodies Across Artistic Modalities

**DOI:** 10.3389/frobt.2020.576790

**Published:** 2021-04-01

**Authors:** Catie Cuan

**Affiliations:** Stanford University, Department of Mechanical Engineering, Stanford, CA, United States

**Keywords:** robotic art, human-robot interaction, performance, improvisation, art installation, motion capture, dance

## Abstract

Millions of industrial robots are used across manufacturing and research applications worldwide. Handfuls of these robots have been used in dance, installation, and theatrical art works as tools and performers. *OUTPUT*, a collaborative artwork presented here, employs an industrial robot as choreographic source material and dancing body in order to reframe these robots as performers and bring them into closer proximity with the general public. This *OUTPUT* work has existed as a performance, installation, and augmented reality application. All three formats of the work include improvisational components, where a human can dance with a representation of themselves alongside an industrial robot, facilitating an embodied and creative experience next to these sequestered machines.

## Introduction

Several million industrial robots operate worldwide today (Heer, 2019). The majority of these robots are used in factories during the manufacturing of many types of products: from cars to consumer electronics. These robots are often inaccessible to the general public, however, because they are regularly large and heavy, at hundreds or thousands of pounds and reaching heights taller than human averages. This size means they are commonly bolted to a single position or track, and thus, cannot be easily removed and transported from their station. In addition, industrial robots are expensive, stiff, and customized to factory settings; they are frequently used for highly precise, repetitive tasks. Finally, they are inaccessible to the general public because in certain cases they do not have force/torque or contact sensors that would indicate whether the robot has hit something unexpectedly, like an obstacle or a person. Therefore, many people have never seen these robots in real life, and even more unlikely, up close.

Artists and researchers have explored how to make these sequestered robots accessible to the general public by incorporating them in artworks, demonstrations, and articles. Some of these artworks explore how industrial robot motion differs from human motion; others include custom software that controls industrial robots through movement (Apostolos, 1985; Byrne et al., 2014; Özen et al., 2017). These industrial robots have also been utilized as characters in plays and installations, in order to prompt imaginings of robot capability.

*OUTPUT*, an artwork created by a dancer and choreographer while in residence at a software engineering company, investigates how to make this unreachable robot presence tangible. The work employs an industrial robot and a human dancer as performers across different mediums - dance, film, and software - in a performance, an art installation, and an augmented reality application. The two primary themes of the work are: 1) Reframe an inaccessible and physically intimidating, yet commonly used, robot into choreographic source material and performance partner. 2) Allow the general public to not only watch but interact and take part in the work by facilitating closeness between humans and robots through many types of media. The work *OUTPUT* tackles questions about how human movements and robot movements become used and reconfigured over time/technology. The work invites the public to explore these questions from an embodied, visceral, choice-making point of view by providing them with improvisational tools.

This article positions *OUTPUT* in relationship to other performances and artworks involving industrial robots. It describes the technical and artistic mechanisms underlying the *OUTPUT* work and how the work extends prior artistic investigations. Following the Introduction, a Background section describes a brief history of industrial robots, prior influential works with industrial robots, and human-robot interaction in relationship to choreography. The Artistic Motivation and Choreographic Execution sections detail the artist’s questions and the mechanics of choreographing the industrial robot. The novel software contributions are recounted in Software Programs. The next three sections: As Performance, As Installation, and As Augmented Reality Application, chronicle *OUTPUT* in each of these forms. Discussion frames within broader theoretical choreographic concepts, the aforementioned prior works, and the questions posed at the conception of the piece. Conclusion describes future directions for the work.

## Background

### Industrial Robot History and Contemporary Context

The Unimate is often considered the first industrial robot arm (Moran, 2007). It was devised in 1959 by George Devol and Joseph Engelberger, two inventors who were deeply fascinated by Isaac Asimov’s robot stories from the early 1940s. The Unimate’s first successful application was as an assembly line robot at the General Motors diecasting plant (Gasparetto and Scalera, 2019). Several industrial robot arms followed as mass manufacturing increased across Japan, Scandinavia, Eastern Europe, and the United States. The Stanford arm was developed by Victor Scheinman in 1969 (Scheinman, 1969), and learnings from this robot informed the design of his PUMA robot arm, an acronym for Programmable Universal Machine for Assembly, built with collaborators at GM and introduced in 1978 (Beecher, 1979). In that decade, new companies came into existence including KUKA, Nachi, Fanuc, Yaskawa, and ASEA; estimates at the time noted a new robotics company was created every month (Conditt, 2011). Since then, industrial robots have expanded across manufacturing applications like welding, packaging, and assembly of items like cars, lumber, and food. According to the International Federation of Robotics (IFR), there are between 2.5 and 3.5 million industrial robots in use today (Heer, 2019). By revenue, ABB Group is the largest creator of industrial robots (Chakravarty, 2019).

Some social or research robots, like SoftBank Robotics’ Pepper, Rethink Robotics’ Baxter, or KUKA’s iiwa, have cameras, collision sensors, and robust readings from force/torque sensors to determine if the robot has made inadvertent contact, causing the robot to slow or stop moving (in some applications, this is also known as active compliance (Fitzgerald, 2013; Pandey and Gelin, 2018). Several historic and contemporary industrial robots are not equipped with such sensors or algorithms to confirm if they have come into contact with their environment as their use does not require contact awareness (Conditt, 2011; Siciliano and Khatib, 2016). This renders industrial robots dangerous for humans in close proximity. As a result, industrial robots are caged or housed in structured, standalone environments away from people. Thus, interactions between humans and these industrial robots are often not directly physical, but rather computational (by programming robot tasks), theoretical (considering other features of the robot, such as its economic, historical, or creative meaning), or through a barrier. An interaction might be fully closed loop or open loop in varying degrees of abstraction.

Discussions about the future of work often include various forms of automation, including industrial robots. These robots are both (human) labor-augmenting and labor-substituting. In a 2017 European Commission study, 72 percent of Europeans believed that robots and artificial intelligence “steal peoples’ jobs.” However, analysis of human employment and robot deployment in Europe does not clearly indicate whether these variables are negatively or positively correlated (Bessen et al., 2020). Analysis on the US Labor Market from 1990 to 2014 indicated that industrial robots had negative effects on employment in localized communities, even though national job figures improved (Acemoglu and Restrepo, 2020). It is not clear whether the actual trend *or the discourse* surrounding contemporary automation is a historical aberration, as one count demonstrates that the majority of today’s jobs did not exist 50 years ago (Atkinson and Wu, 2017; Benanav, 2020). Studies about many types of robots indicate that people who are less familiar with the robotic technology are more likely to fear their impact on employment (McClure, 2018). One analysis argues that humans have a history of projecting their extant fears into fictional representations of robots, which differ significantly from today’s actual robots in research labs and companies (Szollosy, 2017).

### Industrial Robots in the Arts

Creative investigations at the intersection of robotics and various artistic mediums are frequent. Jochum, Millar, and Nuñez drew inspiration from puppetry to formulate strategies for robot motion and design (Jochum et al., 2017). Knight and Gray (Knight and Gray, 2012) drew inspiration from acting, and LaViers, Cuan, Maguire, et al. from dance (LaViers et al., 2018). Researchers also employed the theories of New Animism and its performative technique called “mimesis” to elucidate differences between robot and human entities as a design tool towards building non-anthropomorphic robots (Dörrenbächer et al., 2020).

Decades before the research and artistic works above, in the 1960s, Scheinman collaborated with then Biomedical Engineering PhD student Larry Leifer to create his Stanford industrial robot arm. Leifer became a Professor of Mechanical Engineering at Stanford years after. In the 1980s, dancer Margo Apostolos studied at Stanford for her PhD in physical education and collaborated with Professor Leifer on a series of robot-only ballets, StarDance (1983) and FreeFlight (1984) (Apostolos, 1985). Apostolos began working with Leifer after auditing his course and inquiring why factory robots did not move more gracefully (Williams, 2017). Apostolos also created dances with the Spine industrial robot alongside human dancers in the early 1980s. (Apostolos, 1988).

Prior works with industrial robots led to the formulation of new tools for artists to program robots. Bot & Dolly, a design and engineering studio, buildt software and hardware so artists without robotics experience could interact with industrial robots during the making of films and installations (Byrne et al., 2014). Özen, Tükel, and Dimirovski wrote the program LabanRobot to automatically translate Labanotation into movement for the Mitsubishi RV-7FL (Özen et al., 2017). Researchers engineered an improvising robotic musical instrument that responded to the gestures and sequences played by a human (Hoffman and Weinberg, 2010).

Recording and representing human movement is an ongoing challenge. Choreographers, researchers, and engineers alike have employed notation (such as Labanotation, Eshkol-Wachmann Notation, and Action Stroke Notation (Eshkol et al., 1970; Hutchinson et al., 1977; Badler and Smoliar, 1979; Cooper, 1997) and abstraction (such as stick figures or animations (Marr and Nishihara, 1978; Badler and Smoliar, 1979) to capture and demonstrate motion sequences. Human movement has been utilized as source material for humanoid robots with differing kinematic structures via mappings (Do et al., 2008) and deep learning techniques (Aberman et al., 2020). Industrial robots have appeared in live performances and installations. Two industrial robots appeared with a human actor in the play Fremtiden (The Future), controlled by offstage human operators. Snyder, Johns, Kogan, Avid, and Kilian utilized an industrial robot as a musician during a live performance including projection mapping (Snyder et al., 2015).

### Situating *OUTPUT* Relative to Other Performances and Artworks

To contextualize the *OUTPUT* work, this article includes a detailed discussion of a small number of performance and installation works created with industrial robots. In addition to utilizing an industrial robot in a primarily non-verbal work, these artworks share a few additional themes that will be further addressed in the *OUTPUT* work through the Artistic Motivations section:

- Robot bodies human bodies. As noted, the effect of robot labor is largely hidden inside factories or by the forward passage of time. These prior in closer proximity works, as *OUTPUT* does, attempt to make that robot action known by bringing the robot to the general public.
- Humans as robot creators as well as robot “responders.” The artists utilize the robot as a tool for expression in the making of these works, by modulating the robot’s behavior or movement, and consequently react to that formulated action.

The industrial robots used in these pieces and *OUTPUT* are serial manipulators, meaning one joint is attached in series to a single next joint. This joint can be revolute (revolving around a single axis, like the center point of a clock hand) or prismatic (sliding linearly, like a bead along a string). The last joint of a serial manipulator robot is frequently equipped with a tool or attachment, known as the “end effector” (Siciliano and Khatib, 2016).

In *PROPEL*, Stelarc attached himself to the end effector of an ABB IRB 6640 via a metal bracket and straps in order to feel an intimate connection between himself and the robot. The robot performed a choreographed motion sequence, and due to their physical connection, the robot’s motion dictated Stelarc’s overall trajectory, velocity, position, and orientation in space. The robot’s sphere of motion is constrained by its size, as it was bolted to the floor. The robot’s motors provided the soundtrack. This piece demonstrates an instance of scripting robot motion in order to affect human motion. Stelarc is “stationary” throughout the piece, in that he does not move his own limbs, but is instead directed through space by his attachment to the machine, an instance of human-robot physical coupling. If the robot were to collide with another object while Stelarc was attached, both the robot and Stelarc would be injured. Stelarc relies upon the chosen choreography and the consistency of the robot’s motion in order to guarantee his own safety.

Stelarc’s prior 1995 work, *Ping Body*, is thematically similar. In this piece, he attached a muscle-stimulation system to his right arm and allowed remote audience members to actuate it through their Internet domains. The distance and density of random pinging between these domains and his performance website were mapped to voltages on the stimulation system, forcing Stelarc’s arm to move. This piece demonstrates chaos in both a natural and machine system embodied in one entity - Stelarc retained control over his limbs, head, and torso while allowing the dictation of his right arm (Shanken, 2009; Stelarc, 2009).

In *Black Flags*, Forsythe considered the question “What types of gestures can a robot body perform that a human body cannot?”. He utilized two KUKA industrial robot arms to wave black flags from their end effectors during a 28 min performance. The stationary robots are constrained by their link lengths and confined to a scripted motion. The distributed weight of the flags is prohibitively high for most humans to carry. Forsythe made modifications to the robot’s motion based on the environment when it was reinstalled (Forsythe, 2014; Elkin, 2017). *Black Flags* is not a participatory installation or performance in the same manner as *PROPEL*. It uses a subtler form of robot bodies affecting human bodies as the waving flags create gusts of air that can be felt on the observers’ bodies. In addition, it demonstrates how gestures many be natively generated based on the physical capabilities of the moving body. Once it is scripted, the robot performance does not change each time it is performed. The work is thus shown live to a group of viewers, but is not reactive to the environment or the other robot performer in the pair. Motion consistency is core to the perception of the work.

*Huang Yi and KUKA* is a choreographed dance between several human dancers and a KUKA industrial robot arm. Yi choreographed the robot and also performs in the piece. The robot is affixed with a laser beam in different colors at the end effector, a tactic that creates literal boundaries of space on stage (Kourlas, 2015; Lin, 2016). The scripted robot interacts with Yi in that his movement was initially generated to be a duet with the robot’s. He also physically contacts the robot during their opening duet. Thus, Yi’s interactions with the robot are choreographic and physical. Yi’s movements towards and away from the robot, coupled with his mirroring of the robot’s motions, appear as a shy introduction or manifestation of loneliness. This emotional relationship to the robot paints it as a character. In the closing section, the two dancers’ movements are seemingly dictated by the shifting robot’s moving laser. This evidences the idea of a robot body affecting a human body, now from a physical as well as emotional point of view.

*Mimus* is an installation work with an ABB IRB 6700 robot, also named Mimus by the artist (Gannon, 2017). Eight depth sensors on the ceiling capture the viewers’ moving bodies and software assigns explicit and implicit attributes to them, like age and “engagement level.” The robot reaches towards the “most interesting person” based on those criterion. The robot’s movement is dictated by the commands from the sensing software and a set of behaviors that exude animal behaviors. The robot is stationary on the floor and contained in a glass box (DesignMuseum, 2016; Gannon, 2016). This installation closes the affect loop, in that the robot’s actions affect the installation viewer’s reactions and the installation viewer’s motion influences the robot’s behavior. The encoding of animal-like behaviors again lends character to the robot. While the behaviors are scripted, the sequencing of them is determined by the overall system and therefore unknown in advance.

## Artistic Motivation

The directors of the ThoughtWorks Arts residency held an open call for artists under the title, “Mechanical and Movement,” in spring, 2018. The Consortium for Research and Robotics (CRR), a Pratt-affiliated research institution housed at the Brooklyn Navy Yard, uses two industrial robots for research into materials science, architecture, and human-robot interaction. ThoughtWorks Arts partnered with CRR for this residency and later worked with Red Frog Digital Limited on an overall ThoughtWorks Arts augmented reality application.

*OUTPUT* was created during an initial 12 weeks residency period at ThoughtWorks Arts in New York City over summer, 2018 (Cuan et al., 2019; Cuan, 2020). Additional elements of the work were modified and introduced in fall, 2018, and summer, 2020. The collaborative team included the resident artist (dancer and choreographer Catie Cuan), ThoughtWorks software engineers (Andy Allen, Felix Changoo), ThoughtWorks Arts director Andy McWilliams, CRR roboticists (Gina Nikbin, Noor Saab, Cole Belmont), creative coder Jason Levine, Red Frog Chief Technology Officer Alessandro Mondaini, and ThoughtWorks filmmaker Kevin Barry, with additional creative advising from ThoughtWorks Arts director Ellen Pearlman and CRR director Mark Parsons.

Initial meetings across this collaborative group probed questions of agency and partnership between humans and robots. Cuan identified a central theme of “movement presevation,” or how motions are taught by people to other moving human dancers and translated into directions for robots. The ways in which that motion is altered, glitched, and reformulated became thematic palettes for the work, presenting the questions - what is pure movement? Can aesthetic value be drawn from the records and interpretations of movement rather than the pure, originating movement itself? How do performers, when interacting with their own movement on new bodies at a later time period, own or interpret that motion? How do movement themes, when layered and synchronized across these representations, create a visual group piece, similar to instruments in an orchestra playing in a symphony?

The collaborative team decided to use the ABB IRB 6700 robot named “Wen,” a 10.5 foot high industrial robot located at CRR, as it was a primary example of an inaccessible robot. This Wen robot primarily effects objects and environments through motion and contact (vs. a chat bot that generates readable text). This characteristic makes the robot both a choreographic resource and a viable performer of dance. This robot is used by the team at CRR for materials research and prototyping. The hard materiality of the robot removes it from the realm of science fiction and places it strictly in the present space and moment. Thus a secondary theme emerged of using the robot’s movement quality, appearance, and economic status as choreographic source material. What types of motions does this robot perform in those hard manufacturing scenarios? In what way does changing the context of the presentation of the robot render it as a skilled performer rather than a tool for economic production? Does it alter our impression of repetitive machine motion and perhaps highlight how we ourselves repeat certain motions in order to conform to the machine interfaces around us?

This robot’s enormity and speed renders it hazardous for humans in close proximity similar to other industrial robots described in the Background. This robot is also fixed to its location in the Navy Yard. This absence of tangible physical interaction and mobility led the artistic team to consider other forms of interaction and transportation, a challenge that supported and further extended the theme of recording and reconfiguring motion across distance and body representation.

## Choreographic Execution

Choreographing motion for non-humanoid robots is a challenge extant in the aforementioned works and explored in *OUTPUT*. In order to create *OUTPUT*, the roboticists at CRR shared initial details with the collaborative team about how to program the Wen. Two programming options were possible: selecting a continuous trajectory for the end effector or selecting joint velocities for each separate joint (one at a time, or coupled together). Both of these options force a distinctive choreographic process subject to temporal linearity, meaning a beginning and an end are enforced by each of these programming models. Altering motions once a movement sequence is generated, such as inserting new motions or modifying existing ones, became arduous as the robot’s configuration may result in a singularity, an unsolvable set of joint parameters that cause the robot to stop moving.

Cuan developed a choreographic process where she mapped the robots joints onto select limbs or her entire body. For example, the robot’s end effector might be her head in a full body mapping, or the robot’s end effector may be her hand, in a right arm only mapping. She then created a human dance sequence inspired by the notions of physical labor (watching recordings of the robot moving in a manufacturing context and live at CRR), repetition (as the robot’s motion is frequently repeated during these other manufacturing use cases), and ordered sequencing (for example, the robot’s joints were numbered 1 through 7 in bottom to top order, so runs of joint motions in order might be “2, 3, 4” or “1, 2, 3, 4, 5”). After she created this human dance sequence, she selected when to use full body or isolated mappings on the Wen robot and which joint programming may be suitable for either mapping. Cuan observed the robot performing the sequence and made additions to her own choreography, creating an interactive feedback loop between human and robot body for motion generation. The process of choreographing a 5 min motion sequence onto the robot took approximately 32 h of work at CRR, not including the artist’s time spent generating choreography in advance and between work sessions.

**FIGURE 1:**
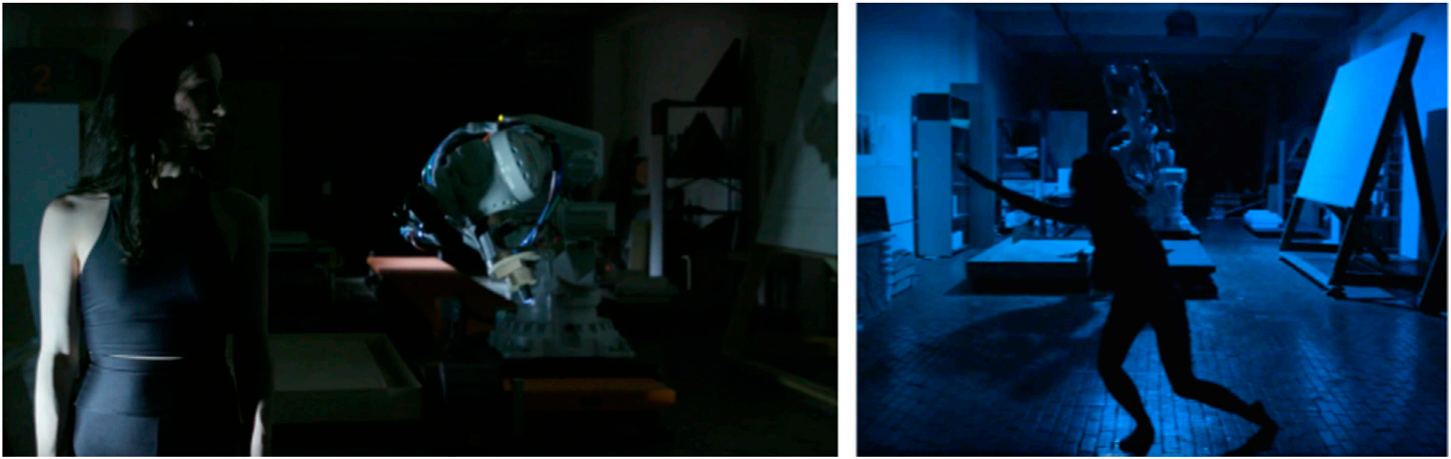
Still images from the *OUTPUT* film. At left, Cuan stands alongside the Wen robot as flood lights illuminate them both. At right, the only light is positioned over the robot, obscuring Cuan’s overall appearance and contrasting obvious elements of the robot and the human - such as number of limbs, joints, and size. Images by Kevin Barry.

The two long motion sequences for human and robot differed on a few dimensions. The human sequence included tempo variability, specific eye gaze points, and broader spatial exploration. The robot was confined to a narrower velocity band and moved along one line, forwards and backwards. At this point, the two long motion sequences for human and robot as well as a generalized mapping process were performance components. The central artistic theme of recording and reinterpreting these sequences across sensing technologies lay ahead. ThoughtWorks engineers Andy McWilliams, Andy Allen, and Felix Changoo extracted the Wen’s joint angle data over the full 5 min sequence and used the angles to populate a moving animation of the robot. Thus, two layers of motion recording and transference existed within the robot animation itself: from Cuan’s original choreography, to the robot’s motions, and finally the resulting joint angles. Cuan and filmmaker Kevin Barry captured footage of the robot moving alongside Cuan’s original performed choreography at the Brooklyn Navy Yard CRR loft, seen in Figure 1. Real time regeneration and repurposing of the animation, robot video footage, human video footage, and human dance would be handled by two new pieces of software and two cameras.

## Software Programs

Two custom pieces of software were written for *OUTPUT*: CONCAT and MOSAIC.

The artist desired the ability to perform the original human choreography next to the translated robot choreography in order to demonstrate the glitches, alterations, and aesthetics of each. For example, a glitch in human choreography might be when the performer loses balance and needs to add an extra step in the sequence. The Wen robot makes no such errors when doing the finished sequence. The human choreography lifts off the floor during jumps, but this trajectory must be altered for the Wen as it is bolted to a track. Given that the robot animation contained two layers of recording translation, while the robot film was one, Cuan also endeavored to show *herself* dancing in layered translation next to these elements. CONCAT was programmed as a result. CONCAT is software built in openFrameworks, a C++ based creative coding platform, that placed a real-time human skeleton captured by a Microsoft Kinect v2 depth sensor next to the 3D animation of the Wen robot. A person oriented towards a laptop or projected screen of CONCAT could see their own skeleton and then, by moving around, observe their captured skeleton interacting with the robot animation through the screen. The Kinect depth sensor’s limited range of capture constrained the interacting person to a particular area. The moving limbs of the robot animation and the captured human skeleton change color according to the fastest moving limb - inherently, in the case of the animation, and dynamically, in the case of the human skeleton. The primary purpose of CONCAT is to allow the participant to try on the robot’s motion.

The inspiration for MOSAIC came from the performer’s initial improvisations with the CONCAT software. Cuan recognized a desire to demonstrate the translation of pure movement across bodies and time in a multiplicative way, such that the prior motions could be contextualized with the real time ones. She envisioned the ability to play multiple instruments in an orchestra simultaneously, similar to a loop pedal or computer music interface, but for dancing bodies. The artist imagined this would secondarily support the question of repetitious motions in a manufacturing context - while a robot in a factory captured over a single time interval might always perform the same motion (i.e. a weld at the same location on a car chassis as the car passes through a factory line every 30 s), the insertion of a real time composer/improviser/conductor like the artist meant select layers and snippets could be arranged into a compelling overall landscape of motion. Cuan began to see this machine labor as possessing meditative continuity rather than monotony and sought to illuminate this reframing of machine labor. In addition, she believed the overall landscape may act as a mirror to the repetitious motions we go through in our own lives, often enforced by technology (typing, door opening, etc.).

**FIGURE 2:**
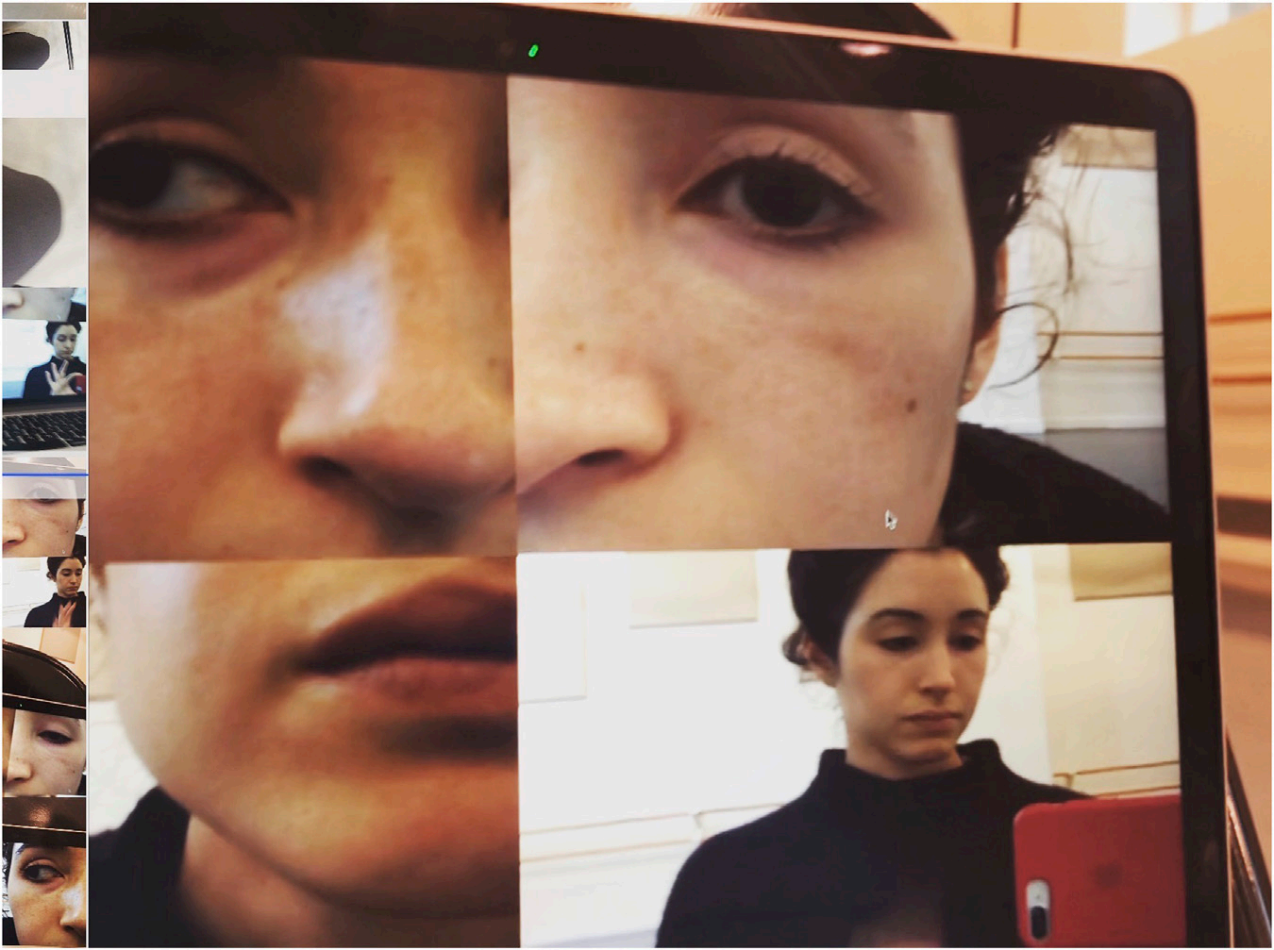
A still image from the MOSAIC software as utilized in rehearsal. The artist began to experiment with proximity in order to exaggerate her features to the scale of the robot’s. Image by Catie Cuan.

MOSAIC is a software built in openFrameworks that stiches together up to 16 moving videos captured from a laptop webcam or external camera source into a single grid/collage. The duration of each individual video is based on a key command from the artist, and the content of each individual video repeats inside its rectangle unless it is removed. A person using the software can add or subtract videos from the collage in order to create a visual quilt of moving bodies. In doing so, the performer can dance with themselves or any other captured bodies in the camera view. MOSAIC additionally allows the artist to alter the size of the moving bodies (via proximity to the camera), supposed physical interaction between the human and the robot (via specific overlapped staging and gestures), number of overall performers (by adding more videos), and audience perspective (by situating the camera at any point on the stage). The orientation of the videos gives the illusion that the bodies are interacting with and affecting each other - for example, a video of the robot moving left to right along an upper left corner square may seem to “bump” the performer if a video Cuan captures on stage where she moves from left to right in the video next to it is timed at the exact right interval. The artist experimented with the MOSAIC tool during rehearsal, as demonstrated in Figure 2.

## As Performance

Over the course of the *OUTPUT* project development, the artist noticed repetitious feelings of being “inside the machine,” as if her own body had become extended into these different devices and other moving bodies. She acutely noticed this when observing the Wen moving through the choreographed sequence for the second time at the CRR loft. During this regurgitation, she felt like her gaze had been transferred into the robot’s end effector and she could see what the robot was “seeing”: details on the ceiling as it tilted upward, the robot’s own arm “elbow” as it rotated, Cuan standing in the Navy Yard studio at one end of the track. Without meaning to, she began marking through the robot’s choreography herself, twisting an ankle or a shoulder as she watched the Wen, as if those robot and human joints were interconnected and the space between herself and the robot”s body had collapsed.

This sensation stretched the initial artistic theme of “pure movement” into one where simultaneous agency and presence is exhibited across recordings and bodies. The capability of the *devices* is de-emphasized while the human body’s capacity for reverberation across modalities of space and time is foregrounded instead. From a choreographer’s perspective, the kernel of humanness - as recognized through shape, proportion, gestural emphasis, and sentimental affect - seemed to proliferate throughout these representations. The symphonic layering, described in this initial artistic theme, could be one of controlled, dictated multiplicity, rather than a byproduct of recording over time. She felt the need to make this explicit in a live performance. Mark Johnson argued that we make meaning out of our thoughts through “a matter of relations and connections grounded in bodily organism-environment coupling,” that sensorimotor activity may be a sort of objective truth for meaning (Johnson, 2008). Cuan’s sensory responses to watching the robot and the representations of herself support the notion that meaning generation and comprehension are visceral, perhaps even moreso with a novel environmental object.

**FIGURE 3:**
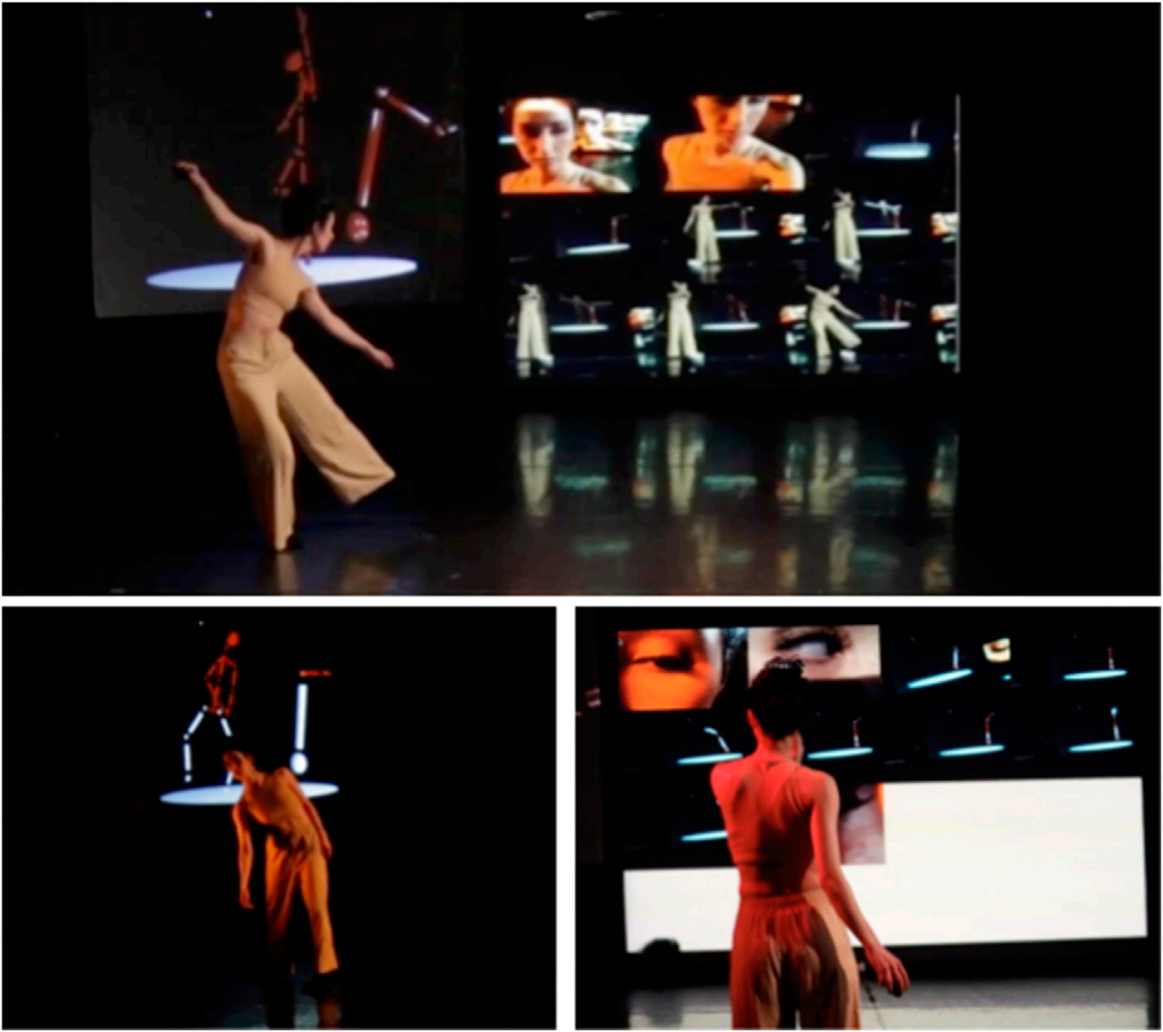
CONCAT and MOSAIC seen during the performance premiere at Triskelion Arts. Cuan uses a wireless mouse to control MOSAIC and orients the web cam in order to capture the projection visuals from both CONCAT and MOSAIC simultaneously to feed into MOSAIC (top). Cuan dances in front of the Kinect depth sensor, populating the human skeleton next to the robot animation on the projector upstage with CONCAT (lower left). The performer constructs a collage of short, captured stage videos with MOSAIC (lower right). Images by Kevin Barry.

Thus, the artist’s aims for a live performance were to generate improvised visual collages of the recorded and live moving bodies in order to illuminate the shared qualities and unique textures of each. She decided to bookend these visual collages with standalone original human dance solos and video of the moving robot performing the “same” sequence in order to highlight each body separately. Cuan was the solo dancer. Both softwares ran live time during the performance on two different laptops, with CONCAT connected to a Kinect and MOSAIC connected to a wired webcam on a 25 foot tether, seen in Figure 3.

Two projectors with screens upstage showed the CONCAT software (with the robot animation and human skeleton) in Figure 3 and the MOSAIC software (as stiched together live time on the tethered webcam) in Figure 3. A wireless keyboard and wireless mouse allowed Cuan to control the MOSAIC software from anywhere on the stage. The webcam on a long tether let her capture her physically present self, the projected animations of the robot and her skeleton in CONCAT, and the projected recorded videos in MOSAIC. This effectively documents what the audience sees on stage during the performance, but from many more proximal and directional angles. The Wen robot could not be transported to the performing space so video of the real robot was shown on a large projected screen. This video footage demonstrates the robot’s scale and original execution of the choreography.

Cuan employed an improvisational modality where she decided how to engage with each software - such as entering the space where the Kinect sensor captured her skeleton with CONCAT, or reorienting the webcam and adding or subtracting videos from the MOSAIC software - over a timed interval between the human dancing solo at the beginning and the standalone robot video at the end. In doing so, Cuan composed live, unique visual collages which conveyed similarities and differences across the live human body, robot animation, and robot film on stage. This practice is akin to live coding, an algorave, or solo dance improvisation. The capture and replay potential does not limit the solo dance improvisation to one body at one time (An algorave is an event where musicians code algorithms in real time on laptops running sound applications, thus producing improvised electronic music. The laptop screen is often projected onto a wall for the audience to observe the programming at the same time as dancing to the music (Collins and McLean, 2014)).

The overall performance lasts between 13 and 15 min and is performed to a single long track of music by artist Bonobo. The mood of the piece is dreamlike, oscillating between wandering and hypnotic, echoing the continuity of dozens of industrial robots bolted along an assembly line. The lighting is used to outline boundaries on stage where the performer will be in front of either of the two cameras - in a recording eligible zone. The performer wears a fleshtoned, closefitting garment to mimic the monochrome of the actual robot body as well as the captured animation and human skeleton (when they are not moving, each animation is completely red).

## As Installation

The artist’s experience choreographing movement for herself and the robot during the making of the work, as well as the feelings of agency and bodily extension into new machines, were sensations she believed stood in contrast to the threatening or fatalistic impressions people often have of fictional robots. In addition, *OUTPUT* in performance provided a rich opportunity for Cuan to see her moving body redesigned by various sensors and algorithms. She was inspired to improvise with these replications because the replications seemed aesthetically and capably different from her own body. Presenting MOSAIC and CONCAT as interactive tools in an installation would allow other individuals to see their own bodies reimagined through various sensing technologies and to personally interact with the Wen robot in a kinesthetic, open-ended manner.

**FIGURE 4:**
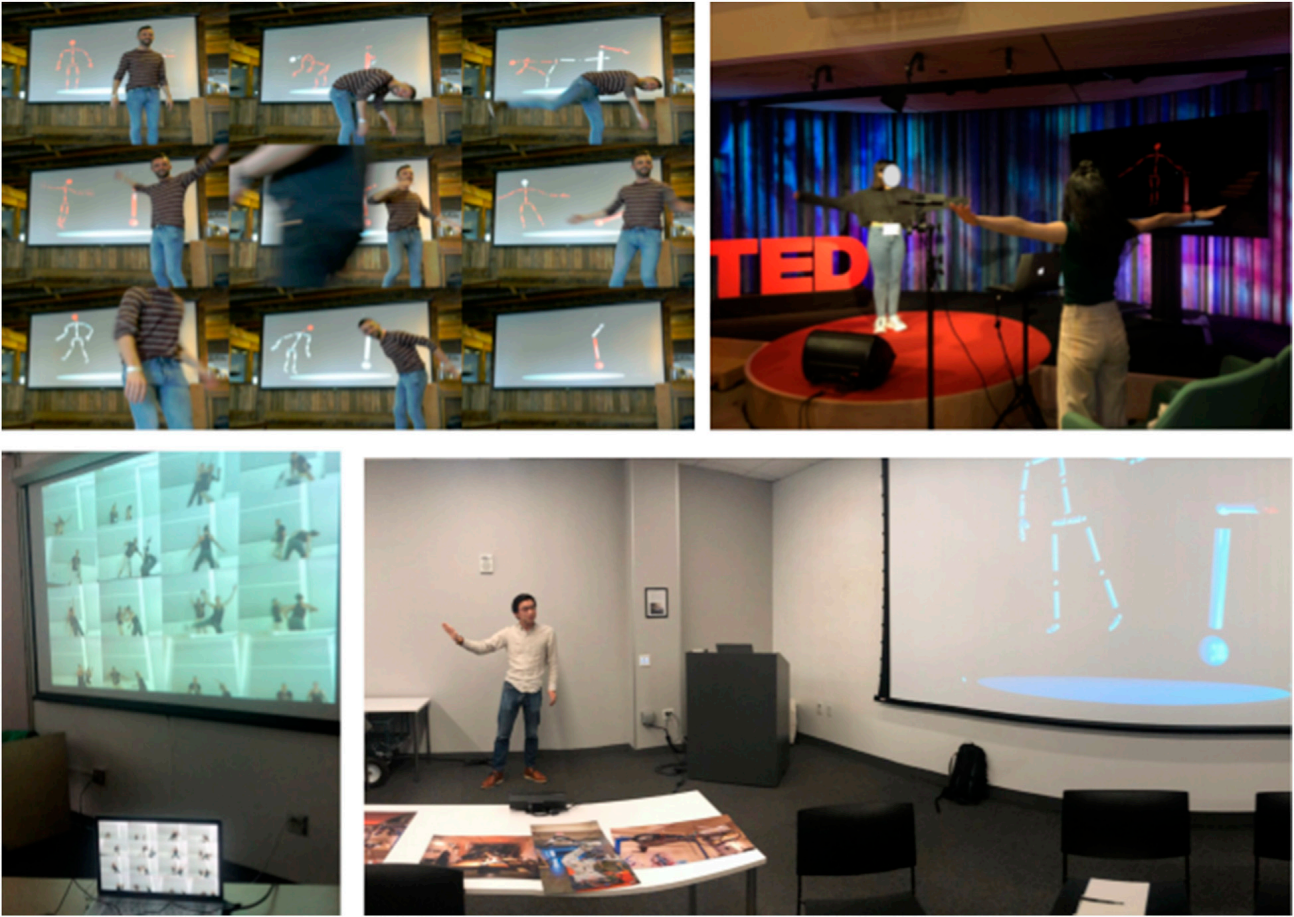
The initial installation showing of *OUTPUT* at Pioneer Works in Brooklyn, New York, in April, 2019 (upper left). MOSAIC and CONCAT run live on two laptops while participants move in front of both the Kinect sensor for CONCAT and webcam for MOSAIC. CONCAT shown as a standalone installation at TED Education Weekend in New York City in February, 2020 (upper right), and at Critical Practices Unit at Stanford University in Palo Alto, in October, 2019 (lower right). MOSAIC shown as a standalone installation at the Dance/USA Conference in Cleveland, Ohio, in June, 2019 (lower left). Images by Catie Cuan and Cameron Scoggins. Used with permission from the participants.

CONCAT and MOSAIC require minimal hardware, only laptops, a Kinect, a webcam, a projector, a screen, and a mouse. CONCAT and MOSAIC have been shown separately and together at five events, totaling approximately 300 participants, across one year. Installation participants have varied in age from toddlers to adults in their 70s. The artist was present at all events and would provide a basic informational script about the software or softwares that comprised the installation. Both tools were demonstrated for the first time as an installation in spring, 2019, pictured in Figure 4. The artist sectioned off a large floor space where the Kinect sensor would detect present bodies. She situated a laptop and a projector around this space, so participants could see their skeleton in one small laptop screen and capture videos of themselves on the second laptop screen. The projector showed CONCAT simultaneously, so passers by could watch someone participating inside the installation and then join themselves. The Wen robot could not be transported from the Navy Yard, thus the scale and size of the robot was diminished in CONCAT. The artist addressed this differential in two ways: by running CONCAT on a large projection screen to make the size of the robot animation as large as possible, and by bringing printed poster-sized photographs of the robot in the CRR loft alongside the choreographer to provide a sense of human-to-robot scale.

Over this yearlong period, participants often shared their verbal reactions with the artist. These installations were not formal experiments, therefore audience reactions were captured through informal artist reflections after the event. When CONCAT was shown, common themes include the surprise at the robot’s small number of joints, curiosity about the appearance of their skeleton in the Kinect representation, and desire to see the “real robot” in person. When interacting with the robot animation through CONCAT, participants would mirror the robot, copy it, try to bump/affect it, and stretch the bounds of their own movement to occupy the entire captured screen. Participants express perceived challenges when trying to mirror or orient themselves in relation to the robot animation in the CONCAT, they ascribed this challenge to the simplicity of the robot or the divergent form factor from their duality (two arms vs. the robot’s single arm, for example). Cuan noticed that CONCAT became a tool for individuals to perform motions that they may not investigate on a regular basis. In doing so, the Wen robot - and by extension the Wen animation - became choreographic source material *for the participants* and the theme of robot motion affecting human motion (as in *PROPEL, Mimus*) was extant in this interaction. CONCAT allows participants to map their own degrees of freedom onto that of the Wen robot’s, posing the question of how their movement is impacted by the vision of a moving industrial robot alongside as well as trying on the Wen’s movement profile. Thus, participants receive kinesthetic insight into the difficulty Cuan faced when formulating motion for a non-anthropromorphic robot. Two showings of CONCAT as installation can be seen in Figures 4.

When MOSAIC was presented as part of the installation, participants expressed detailed observations about their own motion as they viewed squares inside the collage. They also noticed patterns across each square and often generated several collages as they became more familiar with MOSAIC. In instances where they could include the robot animation in the MOSAIC collage, participants frequently captured their skeleton alongside, populating the full collages with not only several squares of video, but all the body representations they could. MOSAIC as a standalone installation has only been shown on one occasion, pictured in Figure 4. When presented individually, CONCAT emphasizes questions of exploratory embodiment and movement influence while MOSAIC underscores recording, repetition, and representation. When presented together, the improvisational and composition aspects of the *OUTPUT* work are reinforced, as the participant is both performer and visual creator of their experience with the Wen robot.

In general, either CONCAT, MOSAIC, or both have been presented in installation form at an adaptable, short term setting, without detailed attention paid to the lighting or exact configuration of the tools. For example, on one occasion CONCAT was shown next to a series of digital musical instruments and at another next to a robotic glove. This removes *OUTPUT* from the realm of performance and somewhat from the realm of installation. An alternative might be similarities to demonstrations or the utilitarianism of a machine on a factory floor. This artistic informality may have led participants to interact more or less freely with the software, or encounter the software’s capabilities with more or less consideration to aesthetics or underlying artistic motivation.

## As Augmented Reality Application

The *OUTPUT* installation experience was extended by introducing another modality - an augmented reality (AR) smartphone application. This application invites individuals to learn about the original *OUTPUT* motivation and artwork and then “Try it yourself.” In the informational section, individuals see a 3D AR rendering of the Wen robot and a video of Cuan dancing alongside, similar to the original performance where the dancer’s and robot’s unaltered bodies were presented at either end of the piece. This information priming about the work parallels Cuan’s spoken introduction at the installation occurrences.

**FIGURE 5:**
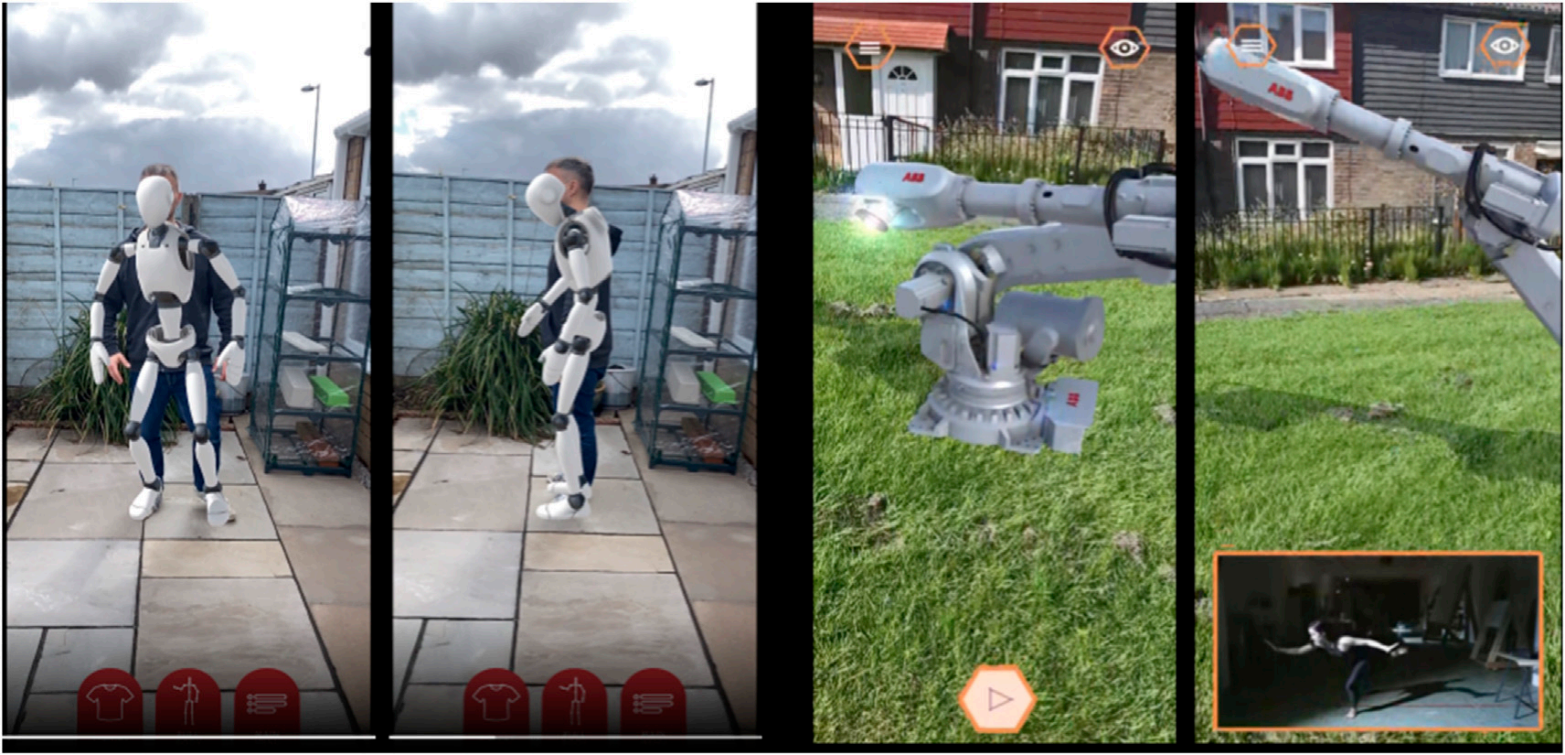
Screenshots from the augmented reality (AR) application in progress. App users can see the ABB robot in 3D through their smartphone camera and the app. The AR robot rotates through poses from the choreographed sequence in the original performance. A video of Cuan dancing alongside the robot appears in an orange overlay on click. The app users can then “Try it yourself” and move in front of the smartphone camera while an overlay resembling a robot follows along their captured motion. Image by Alessandro Mondaini. Used with permission from the participant.

In the “Try it yourself” portion, one individual (the “dancer”) stands with their full body visible to the phone camera and a second individual (the “audience”) films them. The application overlays an animated robot, similar to the robot animation in CONCAT, on top of the dancer’s moving body for the audience member to observe as pictured in Figure 5. As the “dancer” moves, their motion triggers changes in the appearance of the robot overlay (such as color and texture, similar to their captured skeleton in CONCAT), thus inviting them to explore their full range of motion and recognize how their phone’s recording device alters the manifestation of their motion. The “audience” watches these overlay changes in real time, while the “dancer” sees them only during the recording replay. The “dancer” is moving only with the humanoid overlay, rather than the industrial robot, though they can toggle between the Wen robot AR animation and the “Try it yourself” section inside the app.

An option to send their work to the artist appears in the “Try it yourself” section. The works sent to the artist from the application will become elemental moving bodies in future *OUTPUT* performances. This participation practice echoes *Ping Body*, as the full performance system will be altered by the participation of geographically distant application users. In addition, this creates another opportunity for an interactive choreographic loop, where individuals are inspired by the theoretical concepts underpinning the *OUTPUT* work, then record themselves with the robot overlay to be observed by the artist, who will in turn generate new choreography for Wen robot to be incorporated into the next *OUTPUT* performance. This interaction with several individuals across capture modalities and performing bodies is a further reflection of the overall *OUTPUT* artistic motivation.

## Discussion

*OUTPUT* as performance, installation, and augmented reality application investigates two primary artistic motivations: 1) understanding pure movement and technologically-facilitated movement translation, as well as 2) repetitious or industrial objects and movements reframed into a performance context. During the making of the work, the artist recognized additional sentiments of extending into novel machines and how an embodied improvisation alongside these hidden yet ubiquitous robots might encourage individual conclusions about robots. These sentiments arose throughout the collaborative process and led to two original contributions of the work: 1) making a sequestered robot’s physical presence tangible, felt, and known; 2) allowing the public to experience this presence through a variety of improvisational and compositional tools which give them agency to investigate the contrasts between these bodies and their movement profiles again, extending into these novel machines.

Each of the four highlighted prior works as well as *OUTPUT* fall within Lycouris’ “expanded definition of choreography.” Lycouris further described choreography as a practice in which “relationships between all the heterogeneous components of the work can be defined in a coherent manner (Lycouris, 2009).” Each work described in this article includes robots among the “components.” In Stelarc’s *PROPEL*, the “relationship” between the human and robot components is defined mechanically, Forsythe’s *Black Flags* employs a similar mechanical relationship between the flags and the robots. Gannon’s *Mimus*, Huang Yi’s *Huang Yi and KUKA*, and Cuan’s *OUTPUT* define relationship with the robot through scripted motion and responsive behaviors, creating a social, causal relationship between the bodies in the performance/installation. The collection of these relationships and the resulting action between them forms a “compositional meta-system (Lycouris, 2009).” As such, choreography is not only a practice that results in dance, but one that denotes a set of constraints under which motion and action can occur. This necessitates discussion of two further critical concepts: Forsythe’s “Choreographic Object” and Robertson, Lycouris, and Johnson’s approach to “complex systems.”

Forsythe addressed the notion of pure movement in describing his work “Choreographic Objects.” He noted, “But is it possible for choreography to generate autonomous expressions of its principles, a choreographic object, without the body?…A choreographic object is not a substitute for the body, but rather an alternative site for the understanding of potential instigation and organization of action to reside (Spier, 2011).” In all of the aforementioned works, one “alternative site” was a robot body. The scope of the “action” for the robot body varied among the works: Forsythe and Stelarc were primarily interested in dictating the end effector of the robot due to the attachment of the flag/person, whereas Gannon and Yi dictated action for all joints on the whole robot body.

The *OUTPUT* artist changed her interpretation of pure movement at the conclusion of the work. She came to believe that movements or sequences of movements rarely, if ever, have clear origins - does it begin with the idea of the motion? Or when the motion is first done by a body? Or when the inspiration for that motion was first encoded as a vague memory? - and even less clear conclusions - is the motion over when it is performed? Or seen? If it is saved indefinitely in a recording, does the motion ever end? This led her to believe that pure motion has translational and perceptual components - a pure movement is anything which can be recorded and transposed into another body or representation and therefore must be sensed - either by another human or a tool. This conclusion aligns with Forsythe’s assessment that “choreographic objects” can alter the traditionally temporary status of choreography on human bodies, and instead facilitate the existence of a choreographic idea in “another durable, intelligent state (Spier, 2011).”

Robertson, Lycouris, and Johnson describe “complex systems” as “generally diverse and made up of multiple interconnected elements. They are adaptive in that they have the capacity to change and learn from events (Robertson et al., 2007).” The authors further indicate that dance performances with interactive media are an example of such a complex system “in action.” The transference of such a performance into a public space may alter how individuals move or behave within it. Gannon’s *Mimus* and Cuan’s *OUTPUT* as installation are two examples of such “complex systems” and the “interactive media” are robots, animations, and videos. Participants’ motions are captured and become part of the installation in both *OUTPUT* and Gannon’s *Mimus*. Heightened human motions or behaviors result in a more kinetic and possibly compelling installation in both. In contrast to *Mimus*, the *OUTPUT* installation allows participants to see their own motion as captured by the system and modulate it accordingly. In doing so, *OUTPUT* 1) lends an explicit contrast between that of the moving robot and their own motion, and 2) demonstrates how the human fits into and controls elements of the complex system.

Allowing public participants to extend into and “feel like a robot” is part of the secondary contribution of the *OUTPUT* work and probes the motivating question of automated motion. One benefit of this exercise is recognizing the manners in which we ourselves limit, narrow, or mechanize our motions to the requirements of our technologies or the physical tasks in front of us, just as the ABB robot on an assembly line tasked with indenting a sheet of metal or pouring a quantity of silicone. In addition, when the collaborative team - including roboticists and dancers alike - attempted to debug their own work, they were often confronted with the obstacle of the robot’s sensorimotor capabilities being quite different than their own. Gesturing or moving like the robot communicates its limitations to collaborators and also provides clarity on the intended robot trajectory. These limitations are a chasm in public understanding as well: roboticists are starkly aware of the shortcomings in today’s robots, while the public frequently sees edited videos, finalized products, or unilateral success stories. This may lead to inflated expectations of what robots can do, while an embodied personal experience of “feeling like a robot,” through a work like *OUTPUT*, may open the door to an original perspective.

As with many types of artworks, the questions posed by the *OUTPUT* work may not have a singular answer. Audiences and participants described wonderment at their own body’s expressive capacity contrasted by the robot’s limited degrees of freedom. Several individuals noted that dancing with representations of themselves and the robot made them feel multifaceted. Others inquired where the real robot was, as if the true presence of the robot could alter or reaffirm their beliefs about it as viable choreographic source material.

When the augmented reality app launches to the public, participants will film dances of themselves alongside the Wen robot and share them with the artist. This will give them an opportunity to try the human-robot interaction task that the aforementioned artists and Cuan investigated in their works: how the robot body will affect their own.

## Conclusion and Future Work

*OUTPUT* was generated primarily during a 12 weeks residency in summer, 2018, and revisited in summer, 2020. Components of the work have been presented in three formats: as a performance, an installation, and a smartphone application. Two pieces of software, a choreographic process, an improvisational structure, films, and dances were created. The primary artistic motivations were exploration of pure movement across recording mechanisms (and the agency or lack thereof that emerges), as well as the robot’s repetition and utilitarian applications sparking choreographic ideas, variations on a recurrent theme, and participant reflection about interface-enforced repetition in their own lives.

Future instantiations of this work could include surveys or recorded interviews to gauge how audience members interpreted and experienced the fundamental questions around embodiment and robot perception. In order to further explore the public involvement in the work, a permanent or long term home for the *OUTPUT* tools (rather than short term installations that require portability) would permit the artistic team to setup the Wen or a physically similar robot alongside. This fully embodied robot installation would underscore the themes and provide new opportunities for aesthetic and interactive investigation. The *OUTPUT* piece explores some similar themes as other installation and performance works involving industrial robots. *OUTPUT* extends the historical context of industrial robots in performance further by not only bringing an inaccessible robot into close proximity with the public, but also equipping participants with improvisational and directorial tools in nascent mediums (AR) to reevaluate their impressions of themselves and industrial robots.
